# Digital life stories: Navigating together – an observation study of healthcare professionals and people living with dementia

**DOI:** 10.1177/20552076261458019

**Published:** 2026-05-31

**Authors:** Helén Dellkvist, Line Christiansen, Ana Luiza Dallora, Peter Anderberg

**Affiliations:** 1 Department of Health, 4206 Blekinge Institute of Technology, Karlskrona, Sweden

**Keywords:** dementia, digital life story, ethnography, interaction, observation study

## Abstract

**Objective:**

This study, part of a broader project on digital life stories in dementia care, aimed to describe interactions and communication between healthcare professionals and people living with dementia in daycare settings when using digital life stories as a complement to or replacement for written ones.

**Methods:**

With a focused ethnographic approach, 27 observations were conducted in two daycare centres in two municipalities in southern Sweden. The data were analysed in consecutive steps, according to Hammersley and Atkinson.

**Results:**

The observer-identified findings are reported in the following themes: Collaborative engagement; Relational communication, with sub-themes of Verbal communication with visual aids and Non-verbal communication; and Social togetherness.

**Conclusion:**

Digital life stories promote symmetrical, emotionally meaningful interactions by using pictures and profile content to elicit reminiscence and social connection. Interaction quality varies with technological familiarity and group dynamics, underscoring the need for adaptable use. Short, flexible sessions support fluctuating attention, energy, and emotional responses in people living with dementia while enabling spontaneous engagement. Effective implementation requires brief training for healthcare professionals covering technical use, communication strategies, person-centred approaches, and ethical considerations. Portable access and family involvement further strengthen continuity and reduce barriers to integrating digital life stories into routine care.

## Introduction

Globally, more than 55 million people live with dementia, and this number is expected to reach 139 million by 2050.^1^ In Sweden, various types of support in everyday life are tailored to the situation of people living with dementia to maintain quality of life. This applies to specific measures, such as adapting meal situations, providing the option to spend time outdoors, and offering daycare centres.^2^

Spending time at daycare centres is an aid-assessed intervention, meaning people living with dementia can participate in adapted activities during the day. Moreover, daycare activities adapted for people living with dementia emphasise a person-centred approach, fostering a sense of community, helping them remain in their homes, and providing temporary relief to relatives. The activities also contribute to social interaction, structure, and content throughout the day, including meaningful participation with support from Healthcare Professionals (HPs). The design of such activities should help stimulate physical activity and maintain the functional abilities required for daily living.^3^ The activities must be individually designed regarding the degree of dementia. There may be a need for nursing support or a place to meet and discuss issues related to their specific situation, helping them feel a sense of belonging.^4^

Since the 1990s, interest in life story work to support person-centred care for people living with dementia has been increasing. The person-centred approach proposed by Kitwood^5^ implies acknowledging the person through recognition, respect and trust as a unique human being, by personalising care, supporting freedom of choice, engaging in effective communication, building good relationships, and collaborating. In accordance with Kitwood’s approach, a starting point for person-centred care can be the dialogue between the person and the HP, which lays the groundwork for a mutual, respectful partnership that builds on the person’s life narrative.^5^ Life stories often focus on impactful events, allowing the past to be reinterpreted in the present. Narration provides consistency between past and present. Life story documents typically undergo design changes, but the content usually includes information about childhood, family, careers, life highlights, and earlier interests.^6,7^

Life story work has been shown to enhance HPs’ understanding of people living with dementia.^6,8–10^ By integrating personal life stories into care, HPs can support reminiscence-based activities that encourage people to engage in conversations about significant past experiences. Social and healthcare interventions for people living with dementia need to be tailored to each person’s specific conditions and needs.^4^ Life stories offer valuable insights that can guide HPs in planning and delivering care aligned with personal preferences. Life story work is most effective when undertaken as a continuous process involving the person and their relatives.^9,10^ Dementia can lead to unclear speech, reduced vocabulary, and changes in word association patterns, which may challenge mutual understanding in everyday encounters.^11,12^ In this context, communication becomes a crucial prerequisite for person-centred care, as it enables HPs to recognise and respond to a person’s emotions, needs, and perspectives.^12^ Using information from life stories can facilitate communication by helping HPs interpret expressions, initiate meaningful dialogue, and support the person in articulating their experiences. A dialogical approach further strengthens this process by promoting opportunities for the person to share stories, make sense of them, and reflect on their experiences.^13^

One approach to designing and utilising a person’s storytelling is through Digital Life Stories (DLSs), which can be delivered via web-based applications, USB drives, smartphones, and tablets.^14^ They combine personal and generic content, such as photos, music, and videos, making them more interactive than written narratives.^15^ DLS work in care settings is meaningful for people living with dementia, their relatives, and HPs, even in later stages of dementia.^16,17^ Digital technologies also help people living with dementia maintain social contact, feel safe, and remain active in society.^18^ Compared to traditional life stories, DLSs are easier to update and support person-centred care through multimedia integration.

If the life story is digital, it can be easily accessible, combining technology and nursing.^19^ Digital health encompasses a broad field spanning multiple disciplines and leveraging digital technologies.^20^ Technologies have increasingly been used to care for people living with dementia. Still, designs for care settings may face barriers, such as time constraints and physical limitations,^9,19,21–23^ when applied in real-life contexts. Interventions in real-world care settings can help develop an understanding of the relationship between HPs and technology, thereby explaining whether a particular technology is likely to be fruitful for whom and in which contexts.^20^ Research indicates that the use of technology among older people is increasing and that they hold predominantly positive views.^24^ However, a lack of digital skills may limit access to digital tools, exacerbating the digital divide. Adequate training and support must be tailored to local contexts to narrow the digital divide and enable older people’s participation.^21^ To ensure that digital tools become easily accessible in practice, it is essential to provide a thorough introduction to the tool and to offer continuous support as users familiarise themselves with it. In this way, integrating technology into nursing care becomes feasible, as HPs, together with the person, can confidently use digital formats to support person-centred care. In this study, HPs at two daycare centres have been testing DLSs with their guests (people living with dementia) for eight months. This study, part of a broader project on DLSs in dementia care, aimed to describe interactions and communication between HPs and people living with dementia in daycare settings when using DLSs as a complement to or replacement for written ones.

## Methods

### Design

A focused ethnographic approach, which has a holistic view of interaction and communication, was used in this study. This study encompasses verbal and non-verbal communication, as well as social togetherness, within the concept of interaction. The study used episodic participant observations guided by a predetermined research question. In the present study, observation refers to observing a small group of people’s actions in everyday contexts, and interaction refers to both verbal and non-verbal communication. Ethnography focuses on learning *about* people by learning *from* them and experiencing the presence and patterns of community life. It seeks to understand another lifeworld using the self, as much of it as possible, as the instrument of knowing. Collecting and analysing data is a cyclical, grounded process of interaction.^25^ To visualise and gain a sense of the context, Spradley’s^26^ checklist was used, and the data were analysed according to Hammersley and Atkinson’s description.^25^

To observe interactions when HPs and people living with dementia, referred to as *guests*, used a DLS in daycare settings, a digital and web-based tool called Min Memoria (MM), owned and distributed by VilMer, a Norwegian company, was used. MM offers a digitalised version of the life story, making it dynamic and accessible to HPs, guests and their relatives. The person’s life story can be continuously updated, and communication between relatives and HPs could occur on the same platform. Photos and videos could be displayed to the users, facilitating communication.^27^ In this way, the DLS can become a more user-friendly tool for its users. This study was part of a project and did not involve any costs for the daycare centres. Before commencing use of MM, a consultant from the manufacturer, the CEO of Wera Welltech, and the responsible person for disseminating knowledge and practical guidance regarding welfare technology tools such as MM, held introductory meetings (April 2024 and September 2024) attended by HPs, guests, and their relatives. The observational study was introduced, both verbally and in writing, and informed consent was obtained. The consultant assisted the participants in setting up profiles and logging on to MM. Once a month, the research team and the consultant offered digital support meetings for the HPs.

### Settings and participants

A purposeful sample was used for the study. In this study, HPs and guests were the main participants during the observations. The inclusion criteria were as follows: (a) working as an HP at one of the participating daycare centres, (b) being a guest at one of the participating daycare centres, and (c) having tested MM at one of the participating daycare centres. All five participating HPs were women aged 48-59 years. A total of 12 guests, aged 60-87 years, consisting of 8 men and 4 women, participated.

The observations were conducted at two Swedish daycare centres, which were equally comparable in terms of regulations and located in two municipalities in southern Sweden. One daycare centre used a computer in the HP’s office, and the other one utilised iPads for MM use. Several environmental elements contributed to shaping the atmosphere in the settings. Upon arrival, guests were greeted by the welcoming aroma of freshly baked bread and brewed coffee, setting a homely, inviting tone. Everyone was included in the cheerful laughter and small talk between guests and HPs. The ambience was characterised by a sense of safety and comfort, supported by light conversation and quiet tasks carried out in the kitchen. The dining area was adorned with books and flowers, and the furniture created a warm, domestic atmosphere. Before engaging in various activities, such as mini-bus excursions, painting, or crafting within the premises, HPs and guests shared breakfast. Throughout the day, communal meals and coffee breaks encouraged social interaction, concluding in the guests’ return home by taxi. Some interactions took place in an open environment with several guests and HPs at the same table, while others occurred between a single HP and a single guest.

### Data collection

The present study is based on data collected by the first author, who, as a participant observer, entered the environment to understand the culture and routines. The observations occurred during a four-month fieldwork period (October 2024 – January 2025). Data were collected during the daily activities, meals, and coffee breaks. The duration of the observations ranged between 30 and 90 minutes. In total, there were 27 observation sessions. The 12 participating guests attended daycare centres on different weekdays, with some visiting once a week and others 2 to 3 times a week. Consequently, observations were conducted whenever individual guests were present and engaged with MM, resulting in varying numbers of observations per participant. Field notes were continuously written during and after the observations to capture the phenomenon of interest. The field notes documented the context, participants, activities, objects, actions, events, time of day, goals, and emotions that occurred during the study.^26^ The first author observed, while striving to maintain an open mind, and reflected on the emerging moments. Dressed in everyday clothing and using pen and paper instead of digital tools, the observer aimed to blend into the environment. The observer positioned themself close to the guest and HP or stood at a short distance. The latter occurred when observing more than one user simultaneously. Participation included joining activities, setting the table, serving food, and engaging in conversations when appropriate, where building trust was a key responsibility. Some unstructured interviews occurred during the observation sessions, where questions were asked naturally, making the experience a social event. After each observation, the observer spent a moment reflecting on the day with the HPs.

### Data analysis

In ethnography, data analysis is not a distinct stage of the research process. The analysis is continuous and begins partly during data collection, when reflecting on and interpreting fieldnotes, then moves to writing it down, continually aware of how presence and influence may have shaped the data.^25^ The first and second authors analysed the data following Hammersley and Atkinson’s^25^ description. In the first step, they examined how participants performed their activities and interacted to identify patterns of social action and contextual features. This process required the observer to reflect on and interpret the material from both emic and etic perspectives. For example, the observer noted which parts of the DLS the participants used for communication and interaction. The authors organised all field notes - including the observer’s reflections and interpretations - and rewrote them into a document structured by observation. In step 2, the authors reviewed the data and annotated the summarised material with comments on concepts the observer considered relevant to the research objective. For example, they highlighted *pictures for communication* and *body language*. They repeatedly generated and refined concepts as new categories emerged during the sorting and organising of the data. In step 3, the authors clarified the meaning of the concepts and analysed their relationships. They compared each item of data coded within a category, examining similarities and differences between items. This comparison enabled them to refine imprecise categories, split them when necessary, and specify sub-categories. Initially, the authors categorised *pictures for communication* as verbal communication and *body language* as non-verbal communication. Because the data included multiple dimensions, the same type of data appeared in several categories. In step 4, the authors developed emerging concepts into typologies - mutually exclusive sub-categories such as *verbal* and *non-verbal communication*. By specifying the underlying dimensions of these distinctions, they further clarified and modified the categories they had already identified. In step 5, the authors moved iteratively between the data and the emerging concepts, considering plausible alternative interpretations and testing them against the dataset. They ensured the validity of the analysis by triangulating the material and comparing data collected at different times and across the fieldwork phases. For example, they compared data from days with high attendance to days with fewer participants to examine whether interaction patterns, DLS use, and HPs’ support differed across these phases. By cross-checking these observations across varying conditions, they confirmed that the emerging analytical categories remained consistent and did not depend on a single occasion or context. Finally, the co-authors reviewed and validated the analysis results.

### Ethical considerations

Before data collection, the study objective and procedures were presented verbally and in writing to the participants. They were assured confidentiality and informed of their right to withdraw without explanation. Data were collected only from those who voluntarily agreed to participate and provided written consent. The quotes have been translated from Swedish into English by the first author. The study was approved by the Swedish Ethical Review Authority (Dnr 2025-02787-02). All participants provided written informed consent before participating. This study was registered with ClinicalTrials.gov. ID (NCT06337201).

## Results

The observer-identified findings are reported in the following themes: Collaborative engagement; Relational communication, with sub-themes: Verbal communication with visual aids and Non-verbal communication; and Social togetherness.

### Collaborative engagement

Creating a personal profile in MM often required close collaboration and active engagement between HPs and guests. In all observations, the HPs began by introducing MM and acknowledged that guests may have received prior information but might not remember it. Some guests were cautiously optimistic yet expressed limited technological familiarity, stating they lacked the knowledge to use the iPad without support. A demonstration page was displayed, and the HPs assured ongoing support.

The guests provided their names and dates of birth, and together with the HPs, they navigated the calendar interface on the iPad. A photo was taken with the iPad and approved. The HPs asked guests to describe their personalities, occupations, and families. This seemed difficult for the guests to express, as they reflected before responding regarding their personality, ‘One in the crowd, just one in the crowd’ (Guest 11), acknowledging the difficulty of self-description. The HPs assisted in summarising the narrative, which the guests seemed satisfied with.

The HPs explained that MM is dynamic, with the option to add and remove content according to the guest’s wishes and can be continuously updated. Some guests’ voices revealed emotional engagement as they shared their life stories. The HPs strived to keep up and write down what they said, and expressed verbal suggestions for a summary text after affirming how much the guests had to share. When asked about inviting relatives to access and contribute to content, one guest accepted and responded with humour and enthusiasm, ‘I became your coach’ (Guest 10). The interaction gradually became more symmetrical, with the guests showing increased spontaneity and commitment.

During the observed sessions, the guests determined the content of the posts that the HPs wrote down. They often chose their own emojis and captions, reinforcing a sense of ownership and co-creation. One guest, who typically used MM independently at home, emphasised the importance of content that reflected what others needed to know to communicate effectively as the disease progressed. This guest identified a negative aspect of MM, noting that the manufacturer should be aware that when adding family members, it was not possible to choose the order in which they appeared. The guest selected photos from the day’s activity, delegated the upload to the HP, and finally reviewed the result on their mobile phone.

### Relational communication

The HPs regularly initiated conversations about MM content to support verbal communication. It was common for HPs and guests to have coffee while working with MM, which appeared to be both stimulating and distracting. The focus on MM was occasionally disrupted by other people sitting around the table discussing various topics. However, it prompted others to use the tool when they became interested, allowing them to share their stories. When HPs used MM for the first time, focus tended to be on the tool itself rather than on the person. The HPs presented material in MM and suggested posting photos from the day’s activity. Some guests spoke about topics other than the main subject, or were quieter and more passive during the interaction, leading to one-way communication. The interaction consisted of HPs writing and reading posts aloud in MM, while the guests read silently or spelt it out so that they could hear and participate.

The conversations often revolved around text content concerning the person’s life story, such as relatives seeing and commenting on the posts in MM. Even when the HPs initiated the use and writing, the guests were usually involved and engaged in the interaction. They read aloud, reminisced and confirmed that the written text was correct, or the HP verbally initiated the post’s text, ‘A windy day at…’ (HP 4) and asked the guest to complete the rest. They read aloud together, and the guest shared what they remembered from earlier in the day. The HP verbally suggested posts, ‘What do you want to tell your wife about the day?’ (HP 5) and asked the guest if they wanted to add anything else before any text was published. The guest verbally approved or nodded and chose what to publish in MM.

Some of the guests were more verbal when there were only a few people around. The HPs listened attentively and discreetly added their verbal confirmation to ensure they understood what they were communicating, which emphasised how interesting it was to listen to the guests’ story. In several observed sessions, guests began with brief accounts, which HPs skilfully expanded through follow-up questions. In one observed session, the HP and the guest discovered that they had grown up near each other, and the HP was familiar with the school. They found common ground, which positively impacted the interaction and deepened the bond.

#### Verbal communication with visual aids

Photos from daily activities, childhood, and family, as well as interests and habits, showed that the guests’ spontaneity and engagement increased. Photos evoked stronger recognition than text alone, extending narratives beyond the photo. Guests expressed this as, ‘Good to look at and remember what you forget’ (Guest1). HPs facilitated reminiscence by prompting questions about specific photos, such as school memories, which often led to guest-initiated storytelling. They pointed, asked questions, and actively selected and authored content, while HPs assisted with uploading and writing. These photos evoked pride and triggered memories, prompting broader reflections on life experiences, such as occupations, journeys, and shared activities at the daycare centre. This was confirmed by the guests, who expressed, ‘That is correct! That is how it has been’ (Guest 2). Upon seeing personal photos, they responded with joy and vivid storytelling. Photos of pets, especially the service dog, elicited warm emotional facial expressions, prompting further conversation about animals and personal experiences. ‘There are natural smiles for me there… nice day with my friend!’ (Guest 7). The HPs asked questions, and the guests were generally eager to communicate. It was common for the guests to gradually take over the conversation.

Using a digital tool enabled greater flexibility in MM content. When guests expressed discomfort with certain photos, HPs offered reassurance and the option to remove or replace them. Some guests preferred to observe while HPs managed the content, yet still sought involvement. HPs encouraged guests and their relatives to share holiday photos to view at the daycare centre, which appeared to foster connections across generations and countries. ‘It is great to share photos with family in America, and that they can share their photos too’ (Guest 5). In one observation session, a guest shared that some photos had disappeared, expressing uncertainty about using MM without support at home. Family contributions, such as a relative posting a photo, further enriched the experience and stimulated conversation. HPs asked, ‘Do you look at the photos at home?’ (HP 3). The guests often reopened MM at home, alone or with loved ones, and appreciated having all photos included, reflected emotionally upon with, ‘You are doing something good here! It is nice to see the photos with happy smiles. I am not the only one who is happy’ (Guest 3).

The husband of one of the guests had posted a photo of one of their strolls. The guest discussed the photo and its surroundings, highlighting how a photo can evoke laughter. HPs carefully selected pictures to support communication, especially when engagement began to fade. They aimed to steer conversations toward positive themes - such as family, pets, interests, and shared moments like baking or excursions - while remaining sensitive to distressing topics. When such issues arose, HPs listened attentively before gently shifting focus to uplifting content. Throughout, HPs closely observed guests’ reactions, interpreted emotional responses, and encouraged dialogue. Recognition often occurred when HPs identified the guest in a photo, prompting laughter and a sense of connection. The HPs presented photos of the guest to engage them in the conversation, saying, ‘Who is that in the photo?’ (HP 1). The guest often responded, ‘I have been there!’ (Guest 6). The guest might point to a photo and jokingly say, ‘Who is that [laughing], it is me!’ (Guest 8). It was common for them to look at the photos at home, either alone or with their relatives. Guests appreciated and often wanted to include all the photos taken during the day in MM, as it would make their loved ones happy to see how much fun they had.

#### Non-verbal communication

In observed sessions, HPs and guests sat side by side on a sofa or at a kitchen table, fostering interaction through proximity and face-to-face contact. HPs typically held the iPad, ensuring guests could see and participate comfortably.

Guests repeatedly expressed engagement and reminisced through nonverbal cues - such as leaning forward, smiling, nodding, or humming. Non-verbal guests showed enjoyment through facial expressions and gestures. Photos from daily activities consistently elicited positive reactions, with them pointing, covering their mouths in surprise, or uttering brief comments. Touch and eye contact were natural and frequent, reflecting a mutual sense of comfort and trust. Guests might gently touch HPs’ hands or arms to reinforce the connection. HPs maintained eye contact to validate guest engagement, regardless of who was assisting. Even when technical issues arose, the relational bond remained intact. Occasionally, interaction was hindered when HPs held the iPad, preventing the guest from seeing the content. In such circumstances, guests attempted to peek around the device, indicating a desire to participate. In one observed session, the interaction was initially lacking, as the guest sat on the sofa holding a book. The HP looked for photos to post on MM, which piqued the guest’s curiosity.

Smiles and laughter were present when guests viewed posts from loved ones or heard that family members could access MM. HPs aimed to foster pride and joy, attentively responding to their emotional cues. Verbal guests also relied heavily on non-verbal expressions, using gestures and body language to reinforce meaning. Overall, the atmosphere was relaxed and joyful. HPs and guests shared laughter and mutual understanding, with MM serving as a helpful tool for connection, reminiscence, and emotional expression.

### Social togetherness

In several observed sessions, MM use began with light, friendly small talk, which established a relaxed, inclusive atmosphere. MM became an interwoven activity, as small talk among guests around the table often occurred while HPs wrote on their iPads. The interaction sometimes extended beyond the HP and the guest to include other people at the table. At times, guests wanted to share and show photos and videos, which created a sense of community and togetherness. Other guests became curious about what was being said when MM was used and were invited into the conversation. The iPad was passed around so that others could see the photos. For example, a picture of cinnamon buns prompted a discussion about the benefits of cinnamon, with several guests participating.

It could also be a relaxed moment for small talk with another guest, using MM, or for discussing how to spell something to be written in MM. Conversations about animals, nature, and shared interests created a lively atmosphere around the table, allowing guests to take on the role of experts and lead the discussion. Photos in MM helped guests reminisce and continue the conversation. ‘Being together and feeling a sense of belonging with others helps you remember and allows you to try new and challenging things’ (Guest 11). The guests pointed and clicked around the photos and posts in MM, getting to tell and share their narrative. A photo led to a story, a conversation that extended beyond the iPad and MM, as other guests and HPs were invited to participate.

One guest wanted to tell the story behind their wedding photo. The same guest passed around the iPad and showed the others another picture of her and an HP dancing. This interaction led to happy laughter. The guest said to one of the other HPs, ‘Look how we dance!’ (Guest 9). They demonstrated this with their arms and upper body. The guest was very engaged and happy, and the other guests and HPs also showed great joy related to this activity. The guest wanted to show a video of the dance, which they were noticeably proud of. The HP played the video, and the guest sang along, saying they had always loved singing.

## Discussion

The main findings of this study were that MM, as a dynamic tool, enhanced relational communication by integrating photos, videos, and music, while allowing content customisation and continuous updates. It was user-friendly and accessible, even for guests with limited technological skills, allowing them to engage on their own terms.

Observations showed that visual aids in DLS work promoted reminiscence and social interaction. Personal photos, shared between the daycare centre and the guests’ homes, especially those from childhood and family, enhanced recognition, memory recall, and conversation more than text alone, supporting person-centred care. HPs encouraged family members to share texts and pictures with MM for discussions at the daycare centre. Contributions from relatives enriched interactions, and using MM at home could ease pressure because the guest did not have to answer questions about how the day had been, which could be difficult to remember. Using personal items and content in MM could help HPs focus the conversation and care on what was important to the person they were caring for. The importance of photos and personal items in supporting person-centred care, reminiscence, and stimulating communication is observed through various study designs. Memories have been shown to come back more easily when, for example, looking at photos of a loved one. Accessing memories from further back in time can give the present meaning and context, and also a desire to talk about what one remembers.^6,28,29^ Reminiscence therapy, a recognised non-pharmacological approach, uses familiar items, photos, and music to evoke past events.^7^ However, HPs should bear in mind that looking at photos from the past may evoke sad memories and that private and intimate feelings of disclosure may emerge as an uninvited consequence.^30,31^ The findings in this study revealed a desire among HPs that the use of MM should enable a happy and pleasant time to reminisce and share. Because MM content rarely included sad events or pictures, sad memories were rarely evoked. HPs knew most guests well enough to choose content that would appeal to them. Still, HPs remained open to guests who might want to talk about sad memories.

Technology enables the capturing and sharing of life stories, even allowing virtual visits via tablets or VR, offering positive opportunities for reminiscence.^32^ Follow-up questions encouraged detailed storytelling and balanced dialogue. As HPs used open follow-up questions, for example, about pictures on the iPad, the guest was allowed to steer the conversation more. HPs gave the guest the chance to be the main character and expert on the topic, often leading to a more symmetrical conversation. When adding material to MM, HPs focused on ensuring that the guest’s voice was heard and, where possible, that the guest decided the content. Sometimes HPs made various suggestions, such as different pictures, and the guest chose. Sometimes the guest chose emojis that expressed how they were feeling. As found by Grøndahl et al.,^33^ DLSs amplify the voices of people living with dementia, enabling self-expression and helping HPs understand residents and their relatives.

When using a digital tool, HPs and guests had more choices regarding conversation topics, which enhanced the fluidity. An advantage of the technological device was that it could enlarge the photo so everyone could see it clearly. Using DLSs also enabled HPs and guests to remove content no longer desired and to add or change new material, which facilitated person-centred care. Research on communication tools, such as the Computer Interactive Reminiscence and Conversation Aid (CIRCA), is being conducted. It is a digital tool designed to support people with dementia by utilising generic (non-personalised) multimedia content, such as pictures, music, and videos, to stimulate long-term memory and prompt verbal and non-verbal communication.^34,35^ The interaction can take place in a relaxed atmosphere, where music and videos provide opportunities for various types of engagement such as singing, humming and moving.^36^ A comparison between MM and CIRCA reveals two different but complementary perspectives on digital reminiscence. CIRCA has been developed as an intervention tool that uses multimedia stimulation to strengthen conversation and memory access in people living with dementia, even when their biographical background is unknown to HPs.^34^ MM, on the other hand, digitises and structures the person’s life story and serves as a long-term basis for person-centred care, including getting to know the person, where reminiscence is part of a broader care system. In this way, CIRCA represents a generic reminiscence support, while MM represents a biographically individualised care support.

As previous research confirms, the physical setting during DLS use can influence nonverbal communication; face-to-face positioning and proximity enhance interaction, allowing cues such as pointing, smiling, and movement to music.^14,37–39^ Listening and narrating can occur without the use of words.^7^ The observations clearly showed how both verbal and non-verbal communication were promoted and facilitated through the use of MM. Being able to easily scroll between and point to different pictures and text, combined with proximity and eye contact, increased both the guests’ and HP’s engagement. The guest could nod and point to indicate what they wanted to include in the MM and to approve the content. A simple touch and a smile could elicit expressions in speech or body language. However, overuse of DLSs may cause discomfort, so HPs must detect withdrawal signs to avoid pressure.^30^

Rather than distinguishing between verbal and non-verbal communication as separate or hierarchical forms, the observations suggest that engagement in digital life storytelling is multimodal and co-constructed. The use of MM, in combination with the HP’s supportive role, enabled guests to participate in ways that were not dependent solely on verbal ability. Engagement emerged from the interplay among the guest, the HP, and the digital tool, in which different forms of expression carried equal communicative value. This indicates that MM may facilitate inclusive and person-centred interaction by supporting agency across verbal, embodied, and emotional dimensions, rather than privileging one mode of communication over another.

Findings showed community and social togetherness through photo sharing and storytelling, fostering bonds in a person-centred environment. DLSs documented social life and joyful moments, and life story work helped discover activities that promoted communication and memory recall. DLS content improved family interaction, even across distances, as technology removed physical and geographical barriers. Reality can unfold through social interaction, shaped by mutual interpretation.^25^ Even so, shared history does not guarantee shared experiences; diverse perspectives can enrich reminiscence-based and person-centred conversations, allowing the person to position themselves in a context.^34,35^ This was apparent during the observations, as one guest sharing a memory often prompted another to add their perspective. The social interaction appeared to confer a sense of recognition and meaning on lived experiences. This is also in line with research indicating that DLSs, in various forms such as movie-making, the use of devices for digital books, or personal content as text and pictures, can facilitate memory recall and sharing in collaborative settings, potentially protecting against cognitive decline by maintaining social connections and promoting independence. DLSs facilitate opportunities to participate in well-known and new activities. Collaboration with and the development of DLSs can encourage person-centred care.^36,40^

Some distractions occurred during the observation sessions, often due to television or concurrent activities during meals. This might indicate poor inclusion, inadequate introduction to tools, or the severity of dementia. In line with Spradley’s^26^ checklist, the environment affects the actors involved, and activities can be influenced by specific events, such as meals. Perhaps it was distracting that MM was used during meals while various activities happened around the table. Using DLSs seemed to help guests remain central in their lives, engage in activities, and avoid isolation. Welfare technologies support dementia care at various stages, from planning apps to monitoring systems.^4^

The digital guestbook in MM served as a way for HPs and guests to communicate with family and relatives. Distance was no obstacle to staying connected and preventing the person’s social context from decreasing. These aspects are discussed in previous research, such as how digital tools foster new connections, enabling family updates and intergenerational communication, and how iPads facilitate relationships and life story discussions.^13,29,41,42^ Our findings indicated that HPs, with the help of MM, enabled guests to connect the past with the present. Personal material, such as photos, highlighted the *person*, whether the communication was verbal or non-verbal. Research indicates that DLSs help integrate past, present, and future perspectives, thereby enhancing person-centred care by providing a holistic view of the person. Communication difficulties can affect a person’s ability to express day-to-day needs to family and caregivers, which, in turn, can lead to decreased self-esteem and a lower quality of life. DLSs can support people living with dementia by improving communication, memory, and cognition, and assist HPs in fostering person-centred relationships and care; however, further research is needed.^43,44^

### Methodological considerations and limitations

Access to the field was facilitated by the observer’s prior professional experience as a specialist nurse in caring for older people, which may be seen as a potential bias. However, it also contributed to a nuanced understanding of the context and the ability to interpret interactions and routines with sensitivity. This understanding minimised the observer effect, allowing the observer to adapt smoothly to the environment, engage socially, and act flexibly and empathetically with participants. The observer’s sensitive and attentive presence supported trust-building and minimised disruption during observations. According to Hammersley and Atkinson,^25^ participant observation encompasses four levels of participation. The observer participated to some extent in conversations, rather than passively observing. However, this closeness to the field may also introduce interpretative bias; therefore, reflexivity was maintained throughout the study to critically assess the influence of the observer’s position and prior knowledge.

This study was part of a project and did not involve any costs for the daycare centres. At this stage, it was not a question of implementation; rather, the intention was to test DLSs as replacements for, or complements to, written ones. A notable methodological strength of this study was the structured, comprehensive preparation of participants before MM testing. Introduction meetings were held by a consultant from the manufacturer on two occasions, involving HPs, guests, and their relatives. These sessions ensured that all stakeholders were well-informed about the observational study, both verbally and in writing, and provided an opportunity to obtain informed consent in an ethically sound manner. Technical support was available throughout the test period, and no issues were reported.

Data were generated and interpreted through the social interactions among the observer, participants, and the setting. The observer enhanced confirmability using Spradley’s^26^ checklist for writing field notes and the exact data analysis procedure strengthened the study’s reliability. The observer considered time to be an essential aspect, including what had happened earlier in the day, the duration of the social interactions, the presence of distractions, and the amount of time spent in the field.

To ensure quality and rigour, the authors used the Standards for Reporting Qualitative Research (SRQR), which is recommended for research on social interactions and individual experiences in natural settings.^45^ The data were analysed according to Hammersley and Atkinson’s^25^ description, and the results were presented using verbatim quotes. The authors employed triangulation to strengthen the credibility of their findings, continually reviewing the analytical material to ensure its validity and reliability. Respondent validation, as discussed by Hammersley and Atkinson^25^ was not employed, but it may have strengthened the credibility. However, participants could recall inaccurately, as a significant portion of social action occurred unconsciously and left no lasting impression. Therefore, respondent validation may not be trustworthy.

This study has limitations, including its small sample size. Additional participants could have increased the depth and richness of the data. Due to the small sample size, the transferability of the findings cannot be guaranteed. Increasing the number of participants and broadening the geographical spread could have enhanced transferability.

## Conclusion

DLSs facilitate symmetrical, emotionally rich interactions: photos and profile content prompt storytelling, laughter, and social connections. Interactions vary depending on the level of technological familiarity and group dynamics. To support the implementation of DLSs in elder care, DLS sessions should be short and flexible to accommodate fluctuations in attention, energy levels, and emotional responses among people living with dementia. Shorter sessions also allow for spontaneous use - for example, using a single photo to prompt a brief interaction when emotional or social opportunities arise. Further, it is recommended that HPs receive brief training (1–2 h) covering technical operation, communication strategies, including how to avoid dominating the storytelling, for reminiscence and emotional responsiveness. The training should also include person-centred principles and ethical considerations regarding privacy, consent procedures, and upholding the person’s narrative authority. It is an advantage if DLSs are accessible on portable devices, enabling spontaneous use during everyday care encounters. Immediate access increases the likelihood of spontaneous interactions and reduces barriers for HPs. Implementation should also incorporate family involvement, with relatives contributing photos or stories and encouraging them to use MM at home to ensure continuity between home and daycare settings, and to adapt activities to varying levels of digital familiarity among all concerned. Further research is warranted to evaluate the usability and user-friendliness of DLSs. In particular, future studies should explore people living with dementias’ experiences, including how they wish to create, engage with, and utilise such narratives. Gaining insight into their preferences and expectations is essential for ensuring that DLSs are not only accessible but also meaningful and empowering for the intended users.

## Data Availability

The datasets generated during and/or analysed during the current study are available from the corresponding author on reasonable request. The data is not publicly available due to privacy or ethical restrictions.*
